# High Proportion of Intestinal Colonization with Successful Epidemic Clones of ESBL-Producing Enterobacteriaceae in a Neonatal Intensive Care Unit in Ecuador

**DOI:** 10.1371/journal.pone.0076597

**Published:** 2013-10-11

**Authors:** Viveka Nordberg, Arturo Quizhpe Peralta, Telmo Galindo, Agata Turlej-Rogacka, Aina Iversen, Christian G. Giske, Lars Navér

**Affiliations:** 1 Department of Neonatology, Karolinska University Hospital, Stockholm, Sweden; 2 Department of Clinical Science, Intervention and Technology, Division of Pediatrics, Karolinska Institutet, Stockholm, Sweden; 3 Department of Microbiology, Faculty of Medical science, University of Cuenca, Cuenca, Ecuador; 4 Nijmegen International Center for Health Systems Research and Education, Radboud University Nijmegen Medical Centre, Nijmegen, The Netherlands; 5 Department of Microbiology, Tumor and Cell Biology, Karolinska Institutet, Stockholm, Sweden; 6 Department of Clinical Microbiology, Karolinska Institutet, Stockholm, Sweden; Hôpital Robert Debré, France

## Abstract

**Background and Aims:**

Neonatal infections caused by Extended-spectrum beta-lactamase (ESBL)-producing bacteria are associated with increased morbidity and mortality. No data are available on neonatal colonization with ESBL-producing bacteria in Ecuador. The aim of this study was to determine the proportion of intestinal colonization with ESBL-producing *Enterobacteriaceae*, their resistance pattern and risk factors of colonization in a neonatal intensive care unit in Ecuador.

**Methods:**

During a three month period, stool specimens were collected every two weeks from hospitalized neonates. Species identification and susceptibility testing were performed with Vitek2, epidemiologic typing with automated repetitive PCR. Associations between groups were analyzed using the Pearson _X_^2^ test and Fisher exact test. A forward step logistic regression model identified significant predictors for colonization.

**Results:**

Fifty-six percent of the neonates were colonized with ESBL-producing *Enterobacteriaceae*. Length of stay longer than 20 days and enteral feeding with a combination of breastfeeding and formula feeding were significantly associated with ESBL-colonization. The strains found were *E. coli* (EC, 89%) and *K. pneumoniae* (KP, 11%) and epidemiological typing divided these isolates in two major clusters. All EC and KP had *bla*_CTX-M_ group 1 except for a unique EC isolate that had *bla*_CTX-M_ group 9. Multi-locus sequence typing performed on the *K. pneumoniae* strains showed that the strains belonged to ST855 and ST897. The two detected STs belong to two different epidemic clonal complexes (CC), CC11 and CC14, which previously have been associated with dissemination of carbapenemases. None of the *E. coli* strains belonged to the epidemic ST 131 clone.

**Conclusions:**

More than half of the neonates were colonized with ESBL-producing *Enterobacteriaceae* where the main risk factor for colonization was length of hospital stay. Two of the isolated clones were epidemic and known to disseminate carbapenemases. The results underline the necessity for improved surveillance and infection control in this context.

## Introduction

Nosocomial invasive Gram-negative infections are common causes of morbidity and mortality in neonatal intensive care units (NICU). Due to the high incidence of bloodstream infection among preterm neonates, almost all are exposed to empirical antimicrobial treatment. The high antibiotic pressure and, subsequently, emerging extended-spectrum β-lactamase (ESBL)-producing bacteria has become an increasing clinical problem. [1].

Several reports have characterized the epidemiology of nosocomial spread of resistant bacteria and molecular genotyping to trace horizontal spread between neonates is a commonly used technique. Various risk factors for acquisition in the neonate during NICU stay have been identified, such as prematurity (gestational age <37 weeks), length of stay, previous use of antibiotics and absence of breastfeeding.[1]–[9] However, in contexts where the resistant bacteria have reached endemic levels, the dynamics of transmission seem to depend more on the environmental factors in the NICUs.

The clinical relevance of intestinal ESBL-colonization in neonates seems to be important. In studies from Italy, Brazil, Switzerland and Spain, between 12–50% of neonates colonized with ESBL-producing bacteria have developed bloodstream infection with positive blood cultures. [5], [7], [10], [11].

Outbreaks with multidrug resistant bacteria share common characteristics: the time taken to identify the problem, difficulties to identify the source, the time to implement infection control measures and finally eradication of the bacteria. [12] Adequate infection control practice, to limit horizontal spread of the bacteria and an early empirical antibiotic treatment effective against the causative bacteria is largely decisive for the outcome of neonatal septicemia. Treatment failure is associated with increased mortality and higher costs. [7], [13], [14].

The situation regarding colonization pattern and antibiotic resistance in NICUs in Ecuador is largely unknown. Reports from other parts of the region have shown a high frequency of ESBL-producing bacteria as well as limited resources for infection control and antibiotic resistance surveillance. [4], [8], [15] A recently published report from Colombia describes the first observed outbreak of NDM-1 (New Delhi Metallo-betalactamase) producing *K.pneumoniae* in a neonatal ward in South America. [16].

The objectives of this study were to determine the proportion of ESBL-producing *Enterobacteriaceae* in the intestinal flora of neonates at a NICU in Ecuador, to describe their resistance pattern and to study the main risk factors associated with colonization during neonatal intensive care.

## Methods

### Ethics Statement

The study was approved by the Regional Ethical Committee of Cuenca University, Ecuador and the Regional Ethical Committee in Stockholm, Sweden (no.2010/1460-31/4). Written information about the study was given to the parents and verbal consent was obtained and documented in the patients medical record. The ethics committees approved these consent procedures.

### Study Design, Study Setting and Population

We conducted a prospective cohort surveillance study. The setting was a third level NICU at the Hospital Vicente Corral Moscoso, Cuenca, Ecuador. The NICU, which served approximately 400 admissions per year, consisted of four rooms. One intensive care section had a capacity of four neonates and one intermediate care section was divided into one large room with twelve beds and two smaller rooms with four and five beds respectively. The average patient-to-nurse ratio was 7∶1. There were no doors between the rooms. A sink, chlorhexidine/alcohol hand disinfectant and gloves were available in each room. Gloves were not used routinely when in contact with the neonates. Textile gowns were used by the staff members and changed every day. The shortest space between incubators was 75 cm. Parents were not allowed inside the NICU. Mothers were able to leave breast milk in the milk bank in the unit. Ampicillin and gentamicin were the recommended first line therapy in suspected bloodstream infection and were also the most commonly used agents during the study period. Carbapenem monotherapy was seldom used during the study period.

All admitted to the NICU during a 12-week period, 25/01/2011 to 18/04/2011, and who remained hospitalized for at least 24 hours were included in the study. During the NICU stay, demographic, clinical, and microbiological data were prospectively collected. Data regarding potential risk factors for colonization with ESBL-producing *Enterobacteriaceae* at admission was collected, such as maternal antibiotic treatment during pregnancy, place and mode of delivery and the time of rupture of the membranes. Data regarding risk factors associated with colonization acquired during NICU stay such as birth weight, gestational age, sex, APGAR score, length of hospital stay, comorbidity, antimicrobial therapy (ampicillin, gentamicin, ceftriaxone, oxacillin, imipenem, vancomycin, co-trimoxazole, amikacin, fluconazole), central venous and peripheral catheterization, nasogastric and endotracheal tube insertion and type of feeding (i.e., parenteral, breast milk or formula) were analyzed separately.

### Data Collection and Microbiological Testing

Stool specimens were collected every two weeks. Rectal swabs were bedside plated on MacConkey agar containing cefotaxime and ceftazidime 1 mg/L. The concentration was selected as this is the susceptibility breakpoint used by the European Committee on Antimicrobial Susceptibility Testing (EUCAST) 2011 (http://www.eucast.org/fileadmin/src/media/PDFs/EUCAST_files/Disk_test_documents/EUCAST_breakpoints_v1.3_pdf.pdf). [17] Rectal swabbing is a well-known technique to detect GI tract colonization of *Enterobacteriaceae.* [18] The same person collected rectal swabs from all neonates. The intrauterine environment is sterile and the fetus is protected by chorioamniotic membranes. At birth, the gastrointestinal tract is virtually sterile. [19] There was therefore, no rectal swabs taken at admission. The mean time between admission and first surveillance swab was 6.4 days. Primary microbiological examinations were performed at the Department of Microbiology, Medical University of Cuenca and at the PAHO-certified microbiology laboratory of SOLCA, Cuenca. Phenotypic confirmation was performed by the disk diffusion method on Müller-Hinton agar plates with disks containing ceftazidime and cefotaxime +/− clavulanic acid (Beckton Dickinson, BLL). Identified ESBL isolates were sent to the Department of Clinical microbiology, Karolinska University Hospital, Stockholm, Sweden where the species identification and susceptibility test were performed with Vitek2, using EUCAST breakpoints. An earlier described probe-based PCR-assay was used for molecular identification of *bla*_CTX-M,_ and for assignment to distinct CTX-M phylogroups. [20] Semi-automated rep-PCR was conducted with the DiversiLab (DL) microbial typing system (bioMérieux, Marcy l’Etoile, France), as described previously. [21] Multi-locus sequence typing of selected CTX-M producing *K. pneumoniae* isolates was performed according to the guidelines available on the Pasteur Institute MLST website (http://www.pasteur.fr/recherche/genopole/PF8/mlst/Kpneumoniae.html).

### Statistical Analysis

Correlation between groups was analyzed using the Pearson _X_^2^ test and Fisher exact test. A forward step logistic regression model was used to identify significant predictors for colonization with ESBL-producing bacteria. P values <0.05 were considered statistically significant. The analyses were performed using the JMP 9.0.0 software from SAS Institute Inc., Cary, NC, USA and IBM SPSS Statistics 20 software from IBM Corporation, Armonk, New York, USA.

## Results

### Baseline Characteristics

During the study period, 73 neonates were admitted to the NICU and among them, 93% were admitted before 24 hours of age. The infants’ gestational age varied between 29–41 weeks (mean 35.5 weeks) and their birth weight between 500 and 4900 g (mean 1972 g). Days of hospital stay varied between 3 to 61 days (mean 20.6 days). Clinical infections were suspected in 71% (52/73) of the neonates and out of these 51/52 (98%) received intravenous antibiotic treatment during the NICU stay. Characteristics of the infants and risk factors for colonization with ESBL-producing Gram-negative bacteria during NICU care are summarized in Table 1.

**Table 1:** Characteristics of the neonates and risk factors for colonization with ESBL-producing Gram negative bacteria during NICU care.

	No. (%)	Colonized (n = 41)	Not colonized (n = 32)	*P* value* #	*P* value**
Sex				NS	
- male	39 (53.4)	23	16		
- female	34 (46.6)	18	16		
Gestational age, weeks					
>36	31 (42.5)	13	18	NS	
35–36	20 (27.4)	11	9	NS	
32–34	15 (20.5)	11	4	NS	
<32	7 (9.6)	6	1	NS	
Birth weight, gram					
≥2,500	15 (20.5)	4	11	0.02***	
2,000–2,499	10 (13.7)	5	5	NS	
1,500–1,999	33 (45.2)	19	14	NS	
<1,500	15 (20.5)	13	2	0.009	
Length of stay in NICU, days					
1–10	20 (27.4)	6	14	0.0081***	
11–20	22 (30.1)	8	14	0.039***	
21–30	15 (20.5)	12	3	0.045	0.003
>30	16 (21.9)	15	1	0.0005	0.001
Endotracheal tube	20 (27.4)	13	7	NS	
Central venous catheter	5 (6.8)	3	2	NS	
Peripheral venous catheter	72 (98.6)	41	31	NS	
Nasogastric tube	71 (97.3)	41	30	NS	
Parenteral nutrition	21 (28.8)	16	5	0.038	
Breast milk feeding only	28 (38.4)	13	15	NS	
Formula feeding only	19 (26.0)	9	10	NS	
Breast milk and formula feeding	26 (35.6)	19	7	0.048	0.006
Ampicillin/Gentamicin	45 (61.6)	28	17	NS	
Ceftriaxone, days					
0	51 (69.9)	23	28	0.0046***	
1–5	5 (6.8)	4	1	NS	
6–10	10 (13.7)	7	3	NS	
>10	7 (9.9)	7	0	0.016	
APGAR score at 5 min ≤5	6 (8.2)	2	4	NS	
Malformations	2 (2.7)	1	1	NS	

* Univariate analysis.

** Forward step logistic regression.

# In univariate analysis, when more than two categories were represented within a factor, each category was compared to all other categories.

*** Associated with less colonization. NS = not significant.

### ESBL-colonization and Epidemiological Typing of ESBL

**Figure 1:**
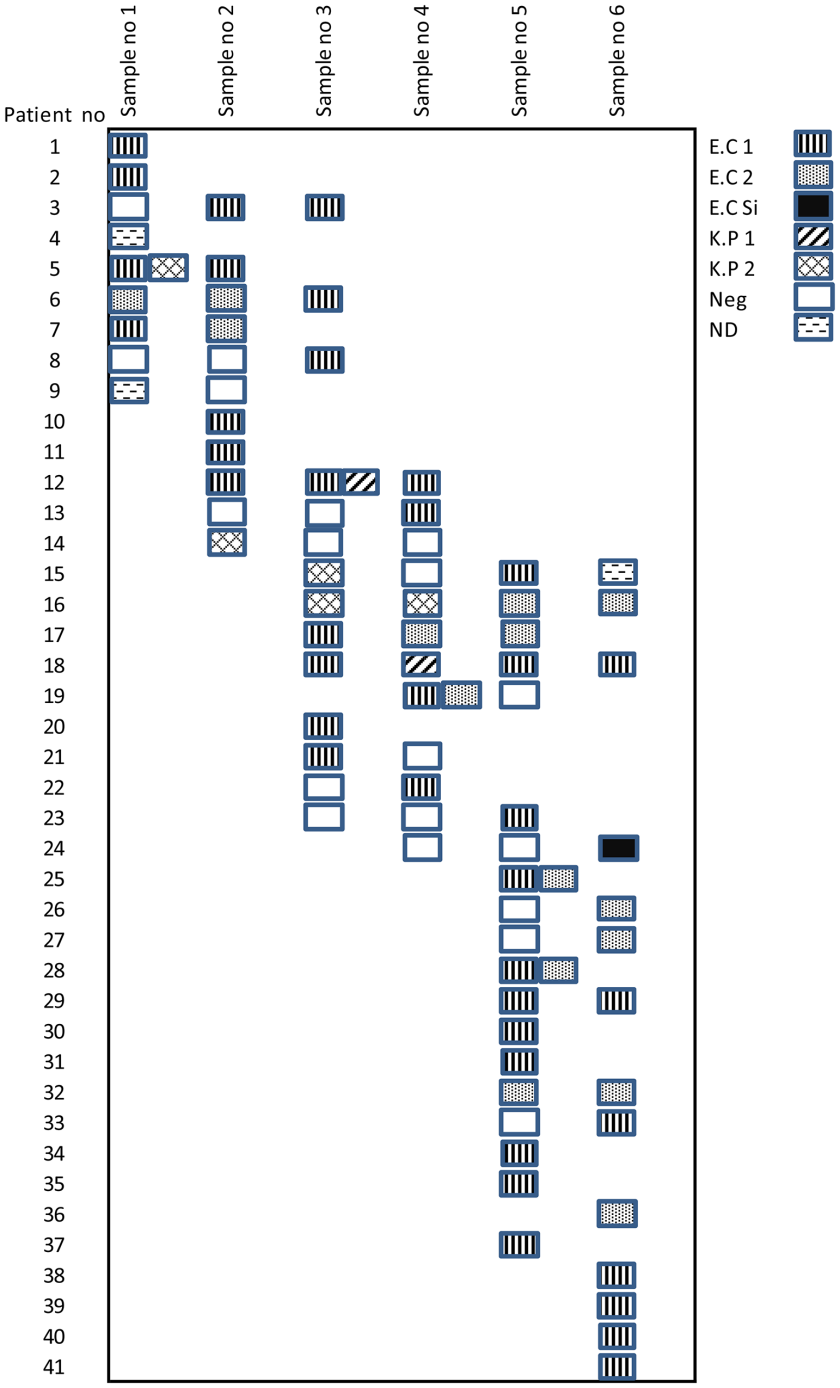
Different strain types of *E. coli* and *K. pneumoniae* isolated from various sampling occasions. E.C = *E.Coli*, K.P = *K. Pneumoniae*, ND = Verified ESBL, DL-typing not done.

In total, 123 specimens were obtained from 73 neonates of whom 41 (56%) were colonized with ESBL-producing bacteria, at some point, during the study period. Of those colonized, 74% (31/41) had a positive stool culture already at study entry (the first rectal swab after admission). Multiple colonization, meaning colonization with two or more DL-types, was found in 27% (11/41), of the neonates. Most neonates were colonized by the same strain for longer intervals and, once acquired; almost all neonates harbored the bacteria until discharge. The majority of the strains were *E. coli* (89%) followed by *K. pneumoniae* (11%). No other ESBL-producing *Enterobacteriaceae* was found. Gentamicin resistance occurred in 98.2% of EC and 100% of KP. Ciprofloxacin resistance occurred in 98.2% of EC and 0% of the KP. All *E. coli*-strains were susceptible to amikacin, unlike the *K. pneumoniae* strains which all were resistant to this antimicrobial. All strains were susceptible to meropenem and imipenem. Epidemiological typing of the ESBL-producing *E. coli* strains identified two clusters and one singleton. The band patterns were compared to those of strains included in the database belonging to the only major successful international epidemic *E. coli* clone ST131. No relation to this clone could be demonstrated. It has previously been shown that ST131 isolates have relatively uniform band patterns when typed with the DiversiLab system. [21] The ESBL-producing *K. pneumoniae* isolates were also divided in two clusters. All *E. coli* and *K. pneumoniae* featured *bla*_CTX-M_ of phylogroup 1, except for the singleton *E. coli* isolate that featured *bla*_CTX-M_ of phylogroup 9. Figure 1 shows different strain types of *E. coli* and *K. pneumoniae* isolated from various sampling occasions. Multi-locus sequence typing was performed on three representatives of each DL-type of the *K. pneumoniae* strains (n = 7) and showed that three (n = 2) belonged to the ST855 lineage and six (n = 5) to the ST897 lineage. Characteristics of the ESBL- producing clones are summarized in Table 2.

**Table 2:** Characteristics of ESBL-producing clones.

Diversilab type		Antimicrobial resistance	Type of CTX-M	Multilocus sequence typing
*E. coli* cluster1	(n = 39)	CTX, CAZ, GEN, CIP	1	NT*
*E. coli* cluster 2	(n = 18)	CTX, CAZ, GEN, CIP, TRI	1	NT*
*E. coli single*	(n = 1)	CTX, CAZ (I), GEN CIP, TRI	9	–
*K. pneumoniae* cluster 1	(n = 2 )	CTX, CAZ (I), GEN, CIP(I), TRI (I), AKN	1	ST 855
*K. pneumoniae* cluster 2	(n = 5)	CTX, CAZ (I), GEN, AKN	1	ST 897

CTX- cefotaxime, CAZ- ceftazidime, GEN- gentamicin, CIP- ciprofloxacin, TRI- trimethoprim, AKN- amikacin.

* NT-MSLT not tested. *E.coli* compared to the clone ST131 and clearly divergent.

### Risk Factors for Colonization with ESBL-producing Bacteria

Several potential risk factors for colonization with ESBL-producing bacteria were analyzed. Risk factors related to the period prior to admission were separately analyzed from those related to the NICU stay. In a univariate model the following potential pre-admission risk factors were analyzed: maternal age <20 years (compared to 20–29 and >29)(NS), maternal age 20–29 (compared to <20 and >29)(NS) and >29 years (compared to <20 and 20–29)(NS), delivery at home (compared to public and private hospital)(NS), delivery at public hospital (compared to at home and private hospital)(NS) and delivery at private hospital (compared to at home and public hospital)(NS), vaginal delivery (compared to Cesarean Section)(NS), rupture of membranes >6 h before delivery (NS), maternal infection or maternal antibiotics during pregnancy (NS). None of these were associated with neonatal colonization with ESBL-producing *Enterobacteriaceae.*

Analyzing NICU associated potential risk factors, a univariate model showed that: birth weight <1,500 g (compared to 1,500–1,999 g, 2000–2499 g, and ≥2,500 g)(OR = 6.96; 95% confidence interval 1.44–33.65; p = 0.009), NICU stay 21–30 days (compared to 1–10, 11–20 and >30 days)(OR 4.0; CI 1.02–15.68; p = 0.045) and >30 days (compared to 1–10, 11–20 and 21–30 days)(OR 17.88; CI 2.21–144.63; p = 0.0005), the use of ceftriaxone >10 days (compared to 0, 1–5 and 6–10 days)(p = 0.016), parenteral nutrition at any time during hospitalization (OR 3.46; CI 1.10–10.83; p = 0.038) and enteral feeding with a combination of breastfeeding and formula feeding (compared to breast feeding and formula feeding)(OR 3.08; CI 1.09–8.72; p = 0.048) were significantly associated with colonization with ESBL-producing *Enterobacteriaceae* (Table 1).

In the univariate model birth weight ≥2,500 g (compared to <1,500, 1,500–1,999 g and 2000–2499 g)(OR 0.21; 95% CI 0.06–0.73; p = 0.02), NICU stay 1–10 days (compared to 11–20, 21–30 and >30 days)(OR 0.22; CI 0.07–0.67; p = 0.0081) and >11–20 days (compared to 1–10, 21–20 and >30 days)(OR 0.31; CI 0.11–0.88; p = 0.039), no use of ceftriaxone (compared to ceftriaxone 1–5, 6–10 and >10 days)(OR 0.18; CI 0.054–0.62; p = 0.0046) were significantly associated with less ESBL colonization (Table 1).

Using a stepwise logistic regression model the length of NICU stay 21–30 days (OR 15.8, CI 2.6–97.5, p = 0.003), >30 days (OR 71.8, CI 6.1–848.4, p = 0.001) and enteral feeding with a combination of breastfeeding and formula feeding (OR 9.5, CI 1.9–46.8, p = 0.006) were significantly associated with ESBL colonization (Table 1).

## Discussion

To our knowledge, this is the first study of neonatal colonization of ESBL-producing bacteria in Ecuador. The main finding was that 56% of the neonates in the NICU acquired intestinal colonization with ESBL-producing *Enterobacteriaceae* during the hospital stay.

In our study, birth weight <1,500 g, parenteral nutrition and the use of ceftriaxone >10 days, but not other antibiotics that were used empirically, were related to increased risk for colonization in a univariate model. These factors are highly related to more severely ill neonates, and thus to the length of hospital stay, and did not persist as independent risk factors in the multivariate analysis. However, the length of hospital stay and enteral feeding with a combination of breastfeeding and formula feeding were defined as independent risk factors for ESBL-colonization.

A number of risk factors for colonization of ESBL producing *Enterobacteriaceae* have previously been proposed. [2], [3], [7], [14] Boo et al. showed, in a case control study that days of hospital stay and early-onset pneumonia were independent risk factors for colonization. In a cohort of 379 neonates, Pessoa et al. found that treatment with a third generation cephalosporin plus an aminoglycoside was an independent risk factor for colonization by ESBL- producing *K. pneumoniae* during the first nine days of hospitalization. [7] Murki et al, confirmed in 2010 that restricted use of cephalosporins in a NICU setting significantly reduced the incidence of ESBL-producing Gram negative bacterial sepsis. [22].

We found two epidemically successful clones of *K. pneumoniae* known for rapid transmission between neonates. The first one, ST855 is a single-locus variant of ST11, and hence part of the putative clonal complex CC11, which also includes the notorious ST258 known to disseminate *bla*_KPC._ The second, ST897 is a single-locus variant of ST14, and hence part of CC14, a clonal complex that has also been associated with dissemination of carbapenemases. The findings of these successful epidemic clones of *Klebsiella* is highly worrisome, as these strains are known to spread very efficiently and it makes the challenge of eradication even greater since these clones are considered harder to eradicate in the NICU environment. [23].

These clones have earlier been detected in Brazil (ST 855) and Canada (ST 897). DiversiLab-patterns for the two main clusters of *E. coli* were compared to patterns in the database for the epidemic ST131 clone, and were found to be clearly divergent. As such, we concluded that neither of the clusters represented ST131, based on previous publications supporting that this clone has a relatively uniform DiversiLab-pattern [21].

The situation of overcrowding, understaffing and poor hand hygiene routines in the NICU increase the risk for transmission and play a major role in the spread of ESBL-producing bacteria. [10] In our study, the association between colonization of ESBL and length of NICU stay, in combination with the finding of epidemic bacterial clusters, makes cross-transmission between neonates the most likely route of colonization, rather than transmission from the mothers, and strengthens the finding of the length of hospital stay as the main risk factor for ESBL-colonization. Infant formula is a well-described pathway for nosocomial infections in NICUs. Human milk has been demonstrated as a protective factor to reduce colonization and infection with resistant *Enterobacteriaceae* in preterm infants. [6] The finding of a combination of formula and breast milk, but not exclusive formula feeding, as a risk factor, was surprising and not in line with previous reports. We have no plausible theory behind this finding and thus, we cannot exclude bias from unknown factors.

The ESBL-producing bacteria, found in our study, were resistant to the preferred first line antibiotic combination in Gram-negative sepsis (Table 2), which makes it probable that the empirical regimen for unknown bloodstream infection might be less effective. Regarding the high level of resistance to ciprofloxacin we can only speculate. It might be explained by a general high consumption of quinolones in the whole population and/or within the hospital as well as colonization of the infant with maternal ciprofloxacin resistant bacteria. There are no other similar studies from Ecuadorian hospitals.

In this type of study it is difficult to determine when/how the colonization occurred. As described in the method part, the gastrointestinal tract at birth is virtually sterile and becomes, gradually, colonized with a variety of ingested environmental and maternal flora. Thus, even if the neonates were colonized prior to admission at the NICU, rectal swabs at admission would most likely have been negative as almost all neonates were admitted within hours from the delivery. How soon colonization can be detected in neonates, after transmission from the mother before admission to NICU is largely unknown. We cannot present any data on the association between colonization and invasive infection as the standard of the microbiological laboratory at the hospital was too poor to deliver reliable data. Amending all problems in the microbiological laboratory at the hospital was beyond the scope and possibilities of this restricted project. Since we can only guarantee the quality of the data obtained from fecal screening we chose not to include data from clinical cultures. One may argue that with such a high frequency of fecal carriage of ESBL-producing *Enterobacteriaceae* it is quite likely that many invasive cases are seen also. Since we have no data from clinical cultures we cannot comment specifically on empirical choice of antimicrobial treatment, other than stating that in general ESBL-producing *Enterobacteriaceae* seem to be very common in this environment. Therefore, the relation between colonization and bloodstream infection in this study is not defined, even though it is likely to involve the same bacteria, since the association between colonization with ESBL-producing strains and clinical significant infections is well described. [5], [7], [10], [11].

One of the main reasons to perform this type of study was to highlight the problem of ESBL-producing *Enterobacteriaceae* in a context where the awareness of this global health issue is very limited. There are several clinical implications of our findings with more than half of the neonates colonized with ESBL-producing bacteria. Firstly, this is to large extent a hidden problem with low awareness among health professionals in most low- and middle income countries, such as Ecuador. As long as no data exists on colonization or resistance patterns, no targeted interventions will be performed. Continuous staff education in the NICU is necessary to limit transmission from care givers. In addition, the local microbiological laboratories need to develop skills, to identify and control outbreaks.

Furthermore, the challenge in these settings will be to find key interventions that are feasible in terms of capacity and costs. There are several well-known measures that can be made to improve infection control without necessarily implying high costs. [24] To limit the transmission of ESBL-producing *Enterobacteriaceae* between neonates, basic procedures of infection control as: appropriate hand decontamination, glove use, appropriate septic practice, isolation strategies and sterilization and disinfection practices would be necessary. However, to implement surveillance systems will mean an enormous challenge to the health system in Ecuador and similar low- and middle income countries.

In conclusion, we found a very high proportion of neonates that were colonized with ESBL-producing bacteria. Risk factors associated with ESBL- colonization were duration of hospital stay and the combination of breast milk and formula feeding. Two successful clones of *K. pneumoniae* were identified. The results demonstrate the necessity for implementing surveillance programs and improved infection control in this context.

## References

[1] Shakil S, Ali SZ, Akram M, Ali SM, Khan AU (2010) Risk factors for extended-spectrum beta-lactamase producing Escherichia coli and Klebsiella pneumoniae acquisition in a neonatal intensive care unit. J Trop Pediatr 56: 90–96. 19608665 10.1093/tropej/fmp060

[2] Bagattini M, Crivaro V, Di Popolo A, Gentile F, Scarcella A, et al (2006) Molecular epidemiology of extended-spectrum beta-lactamase-producing Klebsiella pneumoniae in a neonatal intensive care unit. J Antimicrob Chemother 57: 979–982. 16531430 10.1093/jac/dkl077

[3] Crivaro V, Bagattini M, Salza MF, Raimondi F, Rossano F, et al (2007) Risk factors for extended-spectrum beta-lactamase-producing Serratia marcescens and Klebsiella pneumoniae acquisition in a neonatal intensive care unit. J Hosp Infect 67: 135–141. 17884248 10.1016/j.jhin.2007.07.026

[4] Amaya E, Caceres M, Fanc H, Torres Ramirez A, Palmgren AC, et al (2010) Antibiotic resistance patterns in gram-negative and gram-positive bacteria causing septicemia in newborns in Leon, Nicaragua: correlation with environmental samples. J Chemother 22: 25–29. 20227989

[5] Mammina C, Di Carlo P, Cipolla D, Giuffre M, Casuccio A, et al (2007) Surveillance of multidrug-resistant gram-negative bacilli in a neonatal intensive care unit: prominent role of cross transmission. Am J Infect Control 35: 222–230. 17482993 10.1016/j.ajic.2006.04.210

[6] Duman M, Abacioglu H, Karaman M, Duman N, Ozkan H (2005) Beta-lactam antibiotic resistance in aerobic commensal fecal flora of newborns. Pediatr Int 47: 267–273. 15910449 10.1111/j.1442-200x.2005.02064.x

[7] Pessoa-Silva CL, Meurer Moreira B, Camara Almeida V, Flannery B, Almeida Lins MC, et al (2003) Extended-spectrum beta-lactamase-producing Klebsiella pneumoniae in a neonatal intensive care unit: risk factors for infection and colonization. J Hosp Infect 53: 198–206. 12623321 10.1053/jhin.2002.1373

[8] Cassettari VC, da Silveira IR, Dropa M, Lincopan N, Mamizuka EM, et al (2009) Risk factors for colonisation of newborn infants during an outbreak of extended-spectrum beta-lactamase-producing Klebsiella pneumoniae in an intermediate-risk neonatal unit. J Hosp Infect 71: 340–347. 19147256 10.1016/j.jhin.2008.11.019

[9] Rettedal S, Hoyland Lohr I, Natas O, Sundsfjord A, Oymar K (2013) Risk factors for acquisition of CTX-M-15 extended-spectrum beta-lactamase-producing Klebsiella pneumoniae during an outbreak in a neonatal intensive care unit in Norway. Scand J Infect Dis 45: 54–58. 22991960 10.3109/00365548.2012.713116

[10] Harbarth S, Sudre P, Dharan S, Cadenas M, Pittet D (1999) Outbreak of Enterobacter cloacae related to understaffing, overcrowding, and poor hygiene practices. Infect Control Hosp Epidemiol 20: 598–603. 10501256 10.1086/501677

[11] Valverde A, Coque TM, Sanchez-Moreno MP, Rollan A, Baquero F, et al (2004) Dramatic increase in prevalence of fecal carriage of extended-spectrum beta-lactamase-producing Enterobacteriaceae during nonoutbreak situations in Spain. J Clin Microbiol 42: 4769–4775. 15472339 10.1128/JCM.42.10.4769-4775.2004 PMC522353

[12] Zingg W, Posfay-Barbe KM, Pittet D (2008) Healthcare-associated infections in neonates. Curr Opin Infect Dis 21: 228–234. 18448966 10.1097/QCO.0b013e3282fcec5f

[13] Kaufman D, Fairchild KD (2004) Clinical microbiology of bacterial and fungal sepsis in very-low-birth-weight infants. Clin Microbiol Rev 17: 638–680, table of contents. 10.1128/CMR.17.3.638-680.2004 PMC452555 15258097

[14] Boo NY, Ng SF, Lim VK (2005) A case-control study of risk factors associated with rectal colonization of extended-spectrum beta-lactamase producing Klebsiella sp. in newborn infants. J Hosp Infect 61: 68–74. 15953660 10.1016/j.jhin.2005.01.025

[15] Desimoni MC, Esquivel GP, Merino LA (2004) [Fecal colonization by extended-spectrum beta-lactamase-producing Klebsiella pneumoniae in a neonatal intensive care unit]. Enferm Infecc Microbiol Clin 22: 507–511. 15511390 10.1157/13067617

[16] Escobar Perez JA, Olarte Escobar NM, Castro-Cardozo B, Valderrama Marquez IA, Garzon Aguilar MI, et al (2013) Outbreak of NDM-1-producing Klebsiella pneumoniae in a neonatal unit in Colombia. Antimicrob Agents Chemother 57: 1957–1960. 23357776 10.1128/AAC.01447-12 PMC3623329

[17] European Committee on Antimicrobial Susceptibility Testing, Breakpoint tables for interpretation of MICs and zone diameters. Version 3.1, valid from 2013-02-11.

[18] Lautenbach E, Harris AD, Perencevich EN, Nachamkin I, Tolomeo P, et al (2005) Test characteristics of perirectal and rectal swab compared to stool sample for detection of fluoroquinolone-resistant Escherichia coli in the gastrointestinal tract. Antimicrob Agents Chemother 49: 798–800. 15673772 10.1128/AAC.49.2.798-800.2005 PMC547369

[19] Nair V, Soraisham AS (2013) Probiotics and prebiotics: role in prevention of nosocomial sepsis in preterm infants. Int J Pediatr 2013: 874726. 23401695 10.1155/2013/874726 PMC3557621

[20] Birkett CI, Ludlam HA, Woodford N, Brown DF, Brown NM, et al (2007) Real-time TaqMan PCR for rapid detection and typing of genes encoding CTX-M extended-spectrum beta-lactamases. J Med Microbiol 56: 52–55. 17172517 10.1099/jmm.0.46909-0

[21] Brolund A, Haeggman S, Edquist PJ, Gezelius L, Olsson-Liljequist B, et al (2010) The DiversiLab system versus pulsed-field gel electrophoresis: characterisation of extended spectrum beta-lactamase producing Escherichia coli and Klebsiella pneumoniae. J Microbiol Methods 83: 224–230. 20849889 10.1016/j.mimet.2010.09.004

[22] Murki S, Jonnala S, Mohammed F, Reddy A (2010) Restriction of Cephalosporins and Control of Extended Spectrum Beta Lactamase Producing Gram Negative Bacteria in a Neonatal Intensive Care Unit. Indian Pediatr. 10.1007/s13312-010-0118-y 21048261

[23] Woodford N, Turton JF, Livermore DM (2011) Multiresistant Gram-negative bacteria: the role of high-risk clones in the dissemination of antibiotic resistance. FEMS Microbiol Rev 35: 736–755. 21303394 10.1111/j.1574-6976.2011.00268.x

[24] World Health Organisation, Prevention of nosocomial infections, Practical guide, second edition 2002.

